# Editorial: Grassland-Invertebrate Interactions: Plant Productivity, Resilience and Community Dynamics

**DOI:** 10.3389/fpls.2017.01413

**Published:** 2017-08-15

**Authors:** Michael Rostás, Ivan Hiltpold

**Affiliations:** ^1^Bio-Protection Research Centre, Lincoln University Lincoln, New Zealand; ^2^Hawkesbury Institute for the Environment, Western Sydney University Penrith, NSW, Australia; ^3^College of Agriculture and Natural Resources, University of Delaware Newark, NJ, United States

**Keywords:** biological control, biosecurity, climate change, community ecology, pests

Grasslands are plant communities dominated by non-woody vegetation, in particular species of the Poaceae family. Such communities occur naturally from the tropics to the tundra in areas of low rainfall where soils do not hold enough moisture to support forest growth. Where climatic conditions fail to support natural grasslands, however, they exist due to more or less intensive farming practices and are known as unimproved (= semi-natural) or improved grasslands (Curry, 1994). Grassland types differ significantly in biodiversity and productivity (Kruess and Tscharntke, 2002); the latter being characterized by intensive management, including reseeding, fertilization, and irrigation to encourage the growth of only few plant species with particular value for grazing livestock. Natural and semi-natural grasslands, on the other hand, are often habitats to many rare plant and invertebrate species that depend on no or low-intensity farming, respectively. Natural and anthropogenic grasslands cover more than a quarter of the earth's surface and provide a wealth of ecological services. Grassland ecosystems store a third of the global carbon stocks, ensure water cycling, and are vital for human food production (Gibson, 2009). Invertebrates play major roles in such ecosystems as they contribute to soil fertility, plant growth, pollination, and biological control on the one hand but cause considerable economic loss through herbivory on the other.

This research topic reports new findings and concepts on grassland–invertebrate interactions in semi-natural and improved grasslands with emphasis on the effects of climate change, invasive species, and sustainable control methods of invasive pests. Five reviews, one opinion paper, two methods, and fourteen research articles explore the influence of biotic and environmental factors and management practices on the communities of invertebrates and their relationships with plants and natural enemies. The majority of contributions is dedicated to Australian and New Zealand grassland systems resulting from an invitation to the participants of the ninth Australasian Conference on Grassland Invertebrate Ecology held in Sydney in April 2016. Several studies on invertebrate communities in European grasslands complement our Topic.

*What drives the diversity and distribution of grassland invertebrates and what role do agricultural management practices play?* This question is explored in a number of papers such as the one by Kergunteuil et al. who investigate the nematode fauna in Swiss alpine meadows. Surprisingly, and in contrast to aboveground ecosystems, the study shows that the abundance and diversity of nematodes increases along an elevation gradient, which suggests a more important role for nematodes in the functioning of high-altitude alpine grasslands than previously anticipated. The distribution of soil invertebrates is also the focus of Benefer et al. albeit in low-land permanent pastures dominated by perennial ryegrass (*Lolium perenne* L.). Their work demonstrates that spatial scale is an important factor in describing species distribution. Focussing on the aboveground community of invertebrates, Fournier et al. analyse the rules that determine the co-occurrence network of orthopterans (grasshoppers and crickets) and plants in semi-natural grasslands of the French Jura Mountains. Such networks have a modular structure and the distribution of orthopterans into modules results from trophic and other interactions with plants. The presented models are valuable for biodiversity conservation and will allow for predictions on how such networks will be affected by changes in agricultural practices. Impacts of such practices on grassland invertebrate communities are the focus of a study by Liu et al. who show that herbicide spraying, plowing, and reseeding of permanent grassland can have opposing effects. While herbicide treatment tends to increase soil invertebrates, in particular decomposers due to enhanced food supply, a decrease in abundance can be found after plowing, which constitutes a significant disturbance. Interestingly, most populations were able to recover over a period as short as 1 year after reseeding.

*What are the cascading effects of climate change on invertebrate communities in semi-natural and improved grasslands?* In addition to agricultural intensification, grasslands, and their invertebrate communities need to cope with rising CO_2_ levels, temperature, and changes in precipitation. How prognosed precipitation patterns affect grassland insects is reviewed for the first time by Barnett and Facey. The authors constitute that overall the effects on invertebrates, caused indirectly by changes in plant biomass and diversity, are highly idiosyncratic and dependent on the grassland ecosystem under scrutiny. This conclusion comes with a recommendation for further experiments in this under-studied area of research, which should consider multiple climate factors at the same time, thus reflecting the complex reality of climate change. This notion is reiterated in an Opinion paper by Johnson et al. which furthermore analyses the inherent problem of pseudoreplication in climate change experiments and offers advice on how this can be dealt with. Better results from climate change studies was also the motivation for designing an improved experimental platform to examine ecosystem responses to drought and root herbivory. The so-called DRI-Grass (**D**rought and **R**oot Herbivore **I**nteractions in a **Grass**land) system described by Powers et al. consists of rain-exclusion shelters with a sophisticated irrigation system that measures local rainfall and responds by delivering water in specific proportions of the actual amount with the effect of realistically mimicking natural precipitation patterns. Using the DRI-Grass platform, Torode et al. take a close look at plant and invertebrate community responses, above- and belowground and across a range of expected rainfall scenarios. Their findings suggest that summer drought, in particular, may favor outbreaks of sucking herbivores, probably followed by a density-dependent response in parasitoid abundance. Ryalls et al., in a similar setting, aim at teasing apart the complex interactions between above- and belowground herbivores in a grass-legume model system when exposed to altered precipitation patterns. Interestingly, in this case drought and root damage by weevils lead to decreases in aphid numbers, thus underpinning Barnett and Facey's conclusions about the idiosyncratic and system-specific nature of such interactions.

*How do invasive invertebrates affect grassland diversity and functioning, and how can these invaders be sustainably managed to mitigate their impact on grasslands?* This topic is covered by a third series of papers which focuses in particular on management strategies involving biotic resistance factors. To start with, Frew et al. give an overview of economically important pest species of the Scarab family in Australasia. Their timely review presents basic information on a group of beetles, whose larvae (grubs) cause significant damage by feeding on the roots of pasture plants. A range of abiotic and biotic soil factors as well as plant traits are explored that influence oviposition by adult beetles, larval behavior and survival, and ultimately population dynamics. Three further scarab papers discuss only a single species, the African black beetle *Heteronychus arator*, an invasive grub which is of particular concern to farmers in several countries. Mansfield et al. focus on the dispersal behavior of *H. arator* and address the question why control measures such as insecticide treatment have failed to reduce population levels. Their findings support the hypothesis that African black beetles re-colonize areas of low density by walking and pitfall traps are confirmed as a valuable monitoring tool. An improved method for rearing *H. arator* in the laboratory is presented by Hiltpold et al. which allows *H. arator* to be reared from egg to adult. The protocol may also be useful as a template for rearing other root herbivores, thus facilitating future studies in this important area as research of root–herbivore interactions is often hampered by the availability of the herbivore. Karpyn Esqueda et al. finally give us a broad historical account of improved grassland development in Australia, the establishment of *H. arator* as a major pest and discuss the use of endophytic fungi that produce insect-deterring toxins, as a control method.

Hennessy et al. focus on such grass endophytes (i.e., symbiotic fungi in the genus *Epichloë*) and the impact on insect herbivores. Their chemo-ecological study demonstrates the anti-feedant effect of fungally produced epoxy-janthitrems against caterpillars of *Wiseana* spp. and assesses the role of temperature on the production of these secondary metabolites. Studying several endophyte strains with different metabolic profiles, Popay and Cox confirm the beneficial effects of epoxy-janthitrem producing *Epichloë festucae* Tul. & Tul. against root aphids. Novel endophyte technology, which is based on using selected strains with specific metabolite profiles, is a sustainable control method against insect pests that is well-established in New Zealand and Australia. Bell et al. also highlight the importance of fungi as natural enemies of pasture pests. Using a combination of next-generation sequencing and bioassays, root nematode catching Orbiliomycetes fungi were identified as potential biocontrol agents from suppressive soils. Studies as the above are promising and will ultimately lead to better control of hard-to-tackle soil herbivores.

Adults and larvae of weevils (Curculionidae) are major pasture pests that can feed above- and belowground. In a multi-year study by McNeill et al., the question is raised whether abundance of the host plant *Trifolium repens* L. determines population density of the weevil *Sitona obsoletus* Gmelin. While such a correlation is absent, mortality caused by introduced parasitoids seems to regulate weevil populations. In another weevil study Goldson and Tomasetto aim at elucidating the mechanism behind the observed decline in the initially successful biological control of *Listronotus bonariensis* Kuschel by its parasitoid *Microctonus hyperodae* Loan (Tomasetto et al., 2017). Their laboratory study suggests that the weevil has acquired host plant dependent resistance against its natural enemy, leading to the conclusion that low plant and enemy diversity in agriculture may facilitate the evolution of host resistance. Barratt et al., on the other hand, assess the potential impact of *L. bonariensis*, a pest of improved grasslands, on natural grassland ecosystems and are able to show that the weevil, although present, is not a significant threat to native grass species.

Apart from insects, invasive plants also pose a serious threat to natural and agricultural grasslands, and in many cases specialized herbivorous arthropods such as the thistle leaf beetle, *Cassida rubiginosa* Müller, have been introduced as weed control agents. Cripps et al. examine the evolution of host plant specialization in this beetle and present work that uses a quantitative measure of evolutionary separation between hosts to predict herbivore performance. Studies like these contribute to our understanding of contemporary evolution in novel environments and will aid in predicting non-target risks and host range expansion of biological control agents.

*What makes grasslands resilient to invertebrate threats and community changes?* Two review papers finally highlight the capacity and need for resilience in grassland ecosystems. Moore and Johnson present a thorough survey of the many physical and chemical resistance mechanisms that grasses have evolved against insects and draw our attention to the roots as little is known of belowground defenses in grasses. Strengthening these plant resistance mechanisms in order to deal with new invasive pests is also one of the main recommendations by Goldson et al. Their analysis on pasture biosecurity in New Zealand comes to this conclusion because pre-border controls are deemed less effective for pastures compared with other agricultural sectors due to a range of inherent constraints.

In this Research Topic, we have covered a broad range of themes with an emphasis on the invertebrate fauna of managed but also semi-natural grasslands, the abiotic and biotic factors that affect their dynamics, and some of the control measures that have the potential to provide ecologically and economically sustainable plant protection. Although grasslands are pivotal ecosystems, several aspects of their ecology are still elusive. With increasing anthropogenic pressure on fragile natural and semi-natural grasslands and the need for sustainable management of improved grassland systems, it is of paramount importance to better comprehend their complexity and functioning in order to conserve these resources. We are confident this compilation of papers will be a valuable resource for researchers and others interested in grassland ecology.

## Author contributions

MR wrote the first draft with substantial contributions from IH. Both authors jointly edited successive versions and approved it for publication.

### Conflict of interest statement

The authors declare that the research was conducted in the absence of any commercial or financial relationships that could be construed as a potential conflict of interest.
